# Integrating Bulk Transcriptome and Single-Cell RNA Sequencing Data Reveals the Landscape of the Immune Microenvironment in Thoracic Aortic Aneurysms

**DOI:** 10.3389/fcvm.2022.846421

**Published:** 2022-04-07

**Authors:** Qunhui Wang, Xian Guo, Bo Huo, Xin Feng, Ze-Min Fang, Ding-Sheng Jiang, Xiang Wei

**Affiliations:** ^1^Division of Cardiothoracic and Vascular Surgery, Tongji Medical College, Tongji Hospital, Sino-Swiss Heart-Lung Transplantation Institute, Huazhong University of Science and Technology, Wuhan, China; ^2^Key Laboratory of Organ Transplantation, Ministry of Education, Chinese Academy of Medical Sciences, Wuhan, China; ^3^NHC Key Laboratory of Organ Transplantation, Chinese Academy of Medical Sciences, Wuhan, China; ^4^Key Laboratory of Organ Transplantation, Chinese Academy of Medical Sciences, Wuhan, China

**Keywords:** thoracic aortic aneurysm, immune infiltration, macrophages, T cells, intercellular communications

## Abstract

Thoracic aortic aneurysm (TAA) is a life-threatening cardiovascular disease whose formation is reported to be associated with massive vascular inflammatory responses. To elucidate the roles of immune cell infiltration in the pathogenesis underlying TAA, we utilized multiple TAA datasets (microarray data and scRNA-seq data) and various immune-related algorithms (ssGSEA, CIBERSORT, and Seurat) to reveal the landscapes of the immune microenvironment in TAA. The results exhibited a significant increase in the infiltration of macrophages and T cells, which were mainly responsible for TAA formation among the immune cells. To further reveal the roles of immunocytes in TAA, we inferred the intercellular communications among the identified cells of aortic tissues. Notably, we found that in both normal aortic tissue and TAA tissue, the cells that interact most frequently are macrophages, endothelial cells (ECs), fibroblasts, and vascular smooth muscle cells (VSMCs). Among the cells, macrophages were the most prominent signal senders and receivers in TAA and normal aortic tissue. These findings suggest that macrophages play an important role in both the physiological and pathological conditions of the aorta. The present study provides a comprehensive evaluation of the immune cell composition and reveals the intercellular communication among aortic cells in human TAA tissues. These findings improve our understanding of TAA formation and progression and facilitate the development of effective medications to treat these conditions.

## Introduction

Thoracic aortic aneurysm (TAA) is a life-threatening condition characterized by the dilation of the thoracic artery to a diameter 1.5 times greater than normal size. It is regarded as a silent killer because there are no symptoms unless the individual is suffering from acute aortic events, such as dissection and/or rupture (1, 2). Once TAA ruptures, the first symptom in up to 95% of patients is death with severe pain (3). However, at present, no specific drug therapies have been clinically shown to effectively prevent the progression of TAA except moderate-risk prophylactic surgery (mortality 1–5%), duo to the incomplete understanding of the pathogenesis (4, 5). According to the presence or absence of a syndromic disorder, such as Marfan Syndrome, Loeys–Dietz Syndrome, Ehlers–Danlos Syndrome, or Meester-Loeys syndrome, TAA is broadly categorized as syndromic TAA and non-syndromic TAA with a patient ratio of approximately 2:8 (6, 7). It is well known that syndromic TAA is mainly a consequence of genetic mutations (for example, Marfan syndrome is due to FBN1 mutations, Loeys–Dietz syndrome is due to TGFBR1/2 mutations, and Ehlers–Danlos syndrome is due to COL3A1 mutations) (6). However, non-syndromic TAA results from a variety of etiologies, including hereditary factors, degenerative processes, hypertension, cigarette smoking, and atherosclerotic disease (8). Decades of research on the pathogenesis of TAA has shown that it represents a spectrum of disease pathologies that are the result of complex changes in the cellular and extracellular environment and not a simple degenerative process (8). In particular, immunity and inflammation play important roles in the formation and progression of non-syndromic TAA (9). Therefore, we focused on the landscape of the immune microenvironment in TAAs (refer to non-syndromic TAAs) without congenital diseases in the present study.

Currently, the known molecular mechanisms of TAA caused by inflammation mainly involve three aspects. First, inflammatory responses are involved in endothelial dysfunction. For instance, multiple inflammatory molecules, such as TGFβ, MCP-1, Mst1, and NF-κB, induce endothelial cell (EC) dysfunction through proinflammatory effects and further promote aortic aneurysm formation and development (4, 10, 11). Second, inflammation has been confirmed to induce vascular smooth muscle cell (VSMC) stress, contractile dysfunction, and VSMC phenotypic switching. For example, abnormal activation of the NLR family pyrin domain containing 3 (NLRP3) inflammasome in VSMCs activates caspase-1, which directly cleaves and degrades contractile proteins. This leads to contractile dysfunction, biomechanical failure, and TAA formation (12). In addition, VSMCs lacking SMAD3 switched from a contractile phenotype to a synthetic phenotype (13). Third, inflammation participates in the activation of matrix metalloproteinases (MMPs), leading to alterations in extracellular matrix homeostasis. Activation of STING, a stimulator of interferon genes, induces the MMP-9 production and ECM degradation. It also promotes VSMC apoptosis and necroptosis, resulting in aortic aneurysm/dissection development (14). After decades of accumulated research, we have gained a certain understanding of the roles of inflammation in TAA pathogenesis. However, the failure of the translation of the basic research results to clinical application implicates that the molecular mechanisms of inflammation in TAA remain elusive. Thus, a comprehensive understanding of the landscape of the immune microenvironment in TAA is needed to provide novel insight into the roles of immunity and inflammation.

Hence, in the present study, we downloaded multiple types of datasets, including bulk transcriptomic data and single-cell RNA sequencing (scRNA-seq) data, from the GEO database and adopted various immune-related algorithms (ssGSEA, CIBERSORT, and Seurat) to explore the landscape of immune cell infiltration in TAA at the cell level and in biological processes. Furthermore, intercellular communications among the aortic cells were inferred by the CellChat package. In particular, macrophages were regarded as the most prominent signal senders and receivers in TAA patients and controls, suggesting that macrophages play critical roles in both the physiological and pathological conditions of the aorta. This is consistent with the current state of knowledge. Previous studies only characterized the alterations of immune cell composition. However, we further explored the interactions between immune-immune cells or immune-non-immune cells and clarified the important roles of immune cells, especially macrophages, in TAA pathology. These findings could be helpful in directing future studies aiming to diagnose, treat and prevent TAA-associated complications.

## Materials and Methods

### Transcriptional Data Acquisition

After retrieving the human TAA datasets in the GEO database^1^, we ultimately selected the GSE26155 (15) and GSE155468 (16) datasets for bioinformatics analysis. These datasets contain the largest number of samples among the transcriptome datasets. For the GSE26155 dataset (Platform: GPL5175, Affymetrix Human Exon 1.0 ST Array), we chose 13 normal thoracic aorta samples and 22 uncontroversial TAA samples excluding the samples with bicuspid aortic valve or borderline samples (40 mm < aortic dilation < 45 mm). The GSE155468 dataset [Platform: GPL24676, Illumina NovaSeq 6000 (Homo sapiens)] contains 11 samples (8 TAA samples vs. 3 control samples). It has been reported that all TAA samples are homogenous, and none have hereditary diseases, such as Marfan syndrome, Loeys–Dietz syndrome and bicuspid aortic valve malformation (15, 16). In addition, the normal thoracic aorta tissues obtained from donors (two from recipients) undergoing heart/lung transplantation served as the control group (15, 16). Detailed information on the TAA samples used in the present study is shown in Supplementary Table 1.

### Data Preprocessing of the Microarray Dataset

The raw data of GSE26155 (TAA microarray dataset) were downloaded from the GEO database. Background correction and normalization of the dataset were performed by using the oligo package (version 1.56.0) (17). Principal component analysis (PCA) was performed after data normalization to ensure that the preprocessing analysis was successful. In addition, hierarchical cluster analysis was applied to identify outlier samples by the hclust function in weighted gene co-expression network analysis (WGCNA) (18). Based on the results, two TAA samples (GSM641986, and GSM642067) in GSE26155 were removed from subsequent analyses.

### Identification of Differentially Expressed Genes and Functional Enrichment Analyses

The differentially expressed genes (DEGs) between the control and TAA groups were identified by the limma package (version 3.48.3) (19). Fold change (FC) > 1.5 (|log_2_FC| > 0.585) and adjusted *P*-value < 0.05 were set as the thresholds. The clusterProfiler package (version 4.0.5) (20) was used to perform the Kyoto Encyclopedia of Genes and Genomes (KEGG) pathway enrichment analysis for the identified DEGs, and an adjusted *P*-value < 0.05 (corrected by Benjamini–Hochberg multiple test) was set as the threshold for the enriched significant pathways.

### Estimation of Immune Cell Infiltration

Single-sample gene set enrichment analysis (ssGSEA) can calculate the rank value of each gene according to the expression profile and apply the characteristics of immune cell population expression to individual samples (21, 22). To estimate the population-specific immune infiltration, we performed ssGSEA to derive the enrichment score of immune cells and immune functions for each sample by using the GSVA package (version 1.40.1) (23). A total of 29 immune gene sets from Bindea et al. (24) were obtained; these sets included different immune cells and immune-related pathways and functions. In addition, to validate the effectiveness of ssGSEA, the CIBERSORT algorithm (25)^2^ was applied to calculate the proportion of 22 immune-infiltrating cells for each sample based on the LM22 signature for 100 permutations. The differences in immune cell subtypes between the control and TAA groups were validated again.

### Single-Cell RNA Sequencing Data Analysis

To further validate and explore the landscape of immunocyte infiltration in TAA, GSE155468 (scRNA-seq dataset) was obtained from the GEO database for analysis. The scRNA-seq data were transformed into Seurat objects using the CreateSeuratObject algorithm in the Seurat package (version 4.0.2) (26). Then, the data were filtered with the following criteria: genes detected in >3 cells, cells with >200 distinct genes, and percentage of mitochondrial genes <10%. Next, data normalization and scaling were performed. Then, it was subjected to dimension reduction at three stages of analysis, including the selection of variable genes, principal component analysis (PCA), and uniform manifold approximation and projection (UMAP). Cell clustering was then assessed across a range of predetermined resolution scales to ensure separation of known major aortic cell types without excessive subclustering (resolution = 0.3). Cell types were identified by their expression levels of cell-specific markers according to the CellMarker database (27) (Supplementary Figure 3). Differences in cell proportions between the control and TAA groups were evaluated using the χ^2^ test. DEGs between the control and TAA groups for cell populations of interest were identified by the FindMarkers function in Seurat (26) with log_2_FC > 0.25 and adjusted *P*-value < 0.05 for KEGG pathway analysis by the clusterprofiler package (20). Furthermore, to discover the cell-state transitions from controls to TAA, pseudotime trajectory analysis was performed by the Monocle package (version 2.20.0) (28), which sorted individual cells by DEGs (TAA vs. controls) and constructed a tree-like structure of the entire lineage differentiation trajectory. The profile of DEGs between TAA and controls was calculated by the “differentialGeneTest” function in Monocle.

### Analysis of Cell–Cell Communication

Based on scRNA-seq data, Jin et al. established a signaling molecule interaction database (CellChatDB)^3^ and developed the R package “CellChat” (29). This package is a tool used to infer, analyze and visualize intercellular communication networks (29). Then, the TAA scRNA-seq data were used to explore intercellular communications by applying the CellChat package (version 1.1.3). It can quantitatively characterize and compare intercellular communications through the major referred signaling inputs and outputs for cell populations based on the known structural composition of ligand–receptor interactions. More details about the framework and algorithms of CellChat were described in a previous study (29).

### Immunohistochemical Staining

Thoracic aortic aneurysm specimens (*n* = 6) and normal aortic samples (*n* = 6) were obtained from Tongji Hospital, Tongji Medical College, Huazhong University of Science and Technology. IHC staining was performed on paraffin-embedded sections (5 μm) of the aortic tissues as previously described (30). Briefly, sections were incubated overnight with primary antibodies targeting CD3 (T cell biomarker; 1:2,000, ab16669, Abcam), ITGAM (macrophage biomarker; 1:2000, BM3925, Boster), α-SMA (VSMC biomarker; 1:1,000, ab7817, Abcam), and CD20 (MS4A1, B cell biomarker; 1:1,000, 60271-1-lg, Proteintech), followed by enhancer solution and biotinylated anti-IgG and streptavidin-peroxidase for 30 min at 37°C. Development of the chromogenic color reaction was accomplished using the peroxidase substrate 3,30-diamminobenzidine (Maixin, Fuzhou, China) after sections were washed with phosphate-buffered saline (PBS) three times.

### Statistical Analysis

All statistical analyses were conducted in R software (version 4.1.1). The ssGSEA and CIBERSORT algorithms were used to characterize the immune-infiltrating cells in TAA. The immune infiltration levels and functions between different groups were compared by the Wilcoxon rank-sum test (control vs. TAA) (31). The *P*-value was corrected by the Benjamini-Hochberg multiple test, and *P* < 0.05 was considered statistically significant. Box plots, volcano plots, pie diagrams, and heatmap plots were generated by packages (ggplot2 and pheatmap) provided by the Bioconductor and CRAN projects.

## Results

### Multiple Immune-Related Pathways Involved in Thoracic Aortic Aneurysm

**FIGURE 1:**
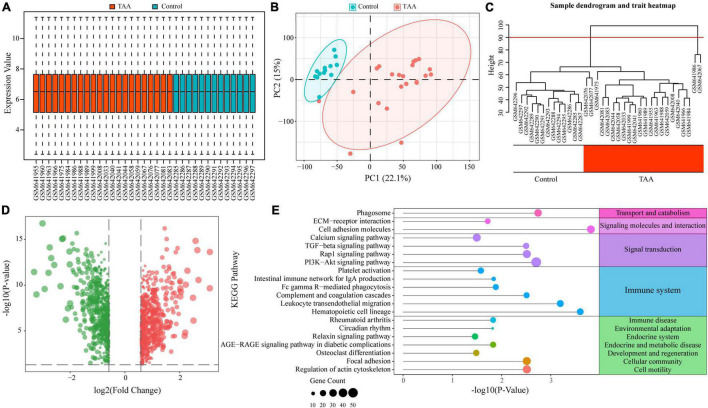
Functional enrichment analysis of differentially expressed genes (DEGs) in thoracic aortic aneurysm (TAA). **(A)** Normalized data of the TAA dataset (GSE26155) are shown in a boxplot. **(B)** Two distinct group-bias clusters were identified by principal component analysis (PCA) based on the TAA dataset. **(C)** Sample clustering was performed to detect outliers. Two samples (GSM641986 and GSM642067) were excluded from the TAA dataset. The red line represents the cutoff of data filtering during data preprocessing. **(D)** DEGs between TAA and controls are shown in a volcano plot. The red and green nodes represent upregulated and downregulated DEGs, respectively. **(E)** KEGG enrichment analysis of the DEGs identified multiple immune-related pathways in TAA.

As previous studies have reported, immune and inflammatory responses play a critical role in TAA. To further investigate the underlying roles of immunity and inflammation in TAA pathogenesis, we first screened the DEGs between TAA patients and control individuals based on TAA microarray data (GSE26155). After data preprocessing, all samples showed a reliable distribution of intensity values and two distinct group-bias clusters between TAA and controls (Figures 1A,B). In addition, two samples (GSM641986 and GSM642067) were excluded from subsequent analyses by hierarchical cluster analysis (Figure 1C). A total of 1525 DEGs in TAA were identified with the threshold of FC > 1.5 and adjusted *P*-value < 0.05, including 769 upregulated DEGs and 756 downregulated DEGs (Figure 1D). The KEGG analysis revealed that the DEGs were enriched in multiple immune-related pathways, such as the TGF-β signaling pathway, hematopoietic cell lineage, leukocyte transendothelial migration, complement and coagulation cascades, Fc gamma R-mediated phagocytosis, intestinal immune network for IgA production, and platelet activation (Figure 1E). The above results confirm the correlation between immune responses and TAA and suggested that a comprehensive understanding of the immune microenvironment will provide more insights into the formation and progression of TAA.

### The Evaluation of Immune Cell Composition in Thoracic Aortic Aneurysm

**FIGURE 2:**
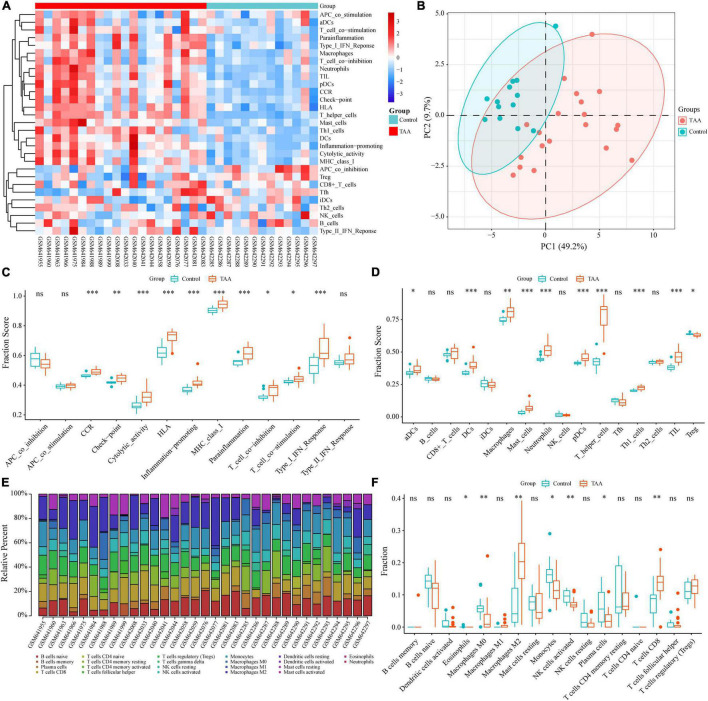
Identification of the infiltrating immune cells and immune functions in TAA. **(A)** Twenty-nine immune-related gene sets, including 16 immune cells and 13 immune processes, were enriched by ssGSEA. **(B)** Two distinct group-bias clusters were identified by PCA based on the 29 immune-related gene sets. **(C,D)** The fractions of immune processes **(C)** and immune cells **(D)** between TAA and control samples are shown in boxplots. **(E)** The fractions of 22 types of immunocytes in each sample were identified by the CIBERSORT algorithm and are shown in the histogram. **(F)** The differences in immune infiltration levels between TAA and control samples, based on the CIBERSORT algorithm, are shown in a boxplot. ns: no significance; *P < 0.05; **P < 0.01; ***P < 0.001.

To reveal the landscape of immunocyte infiltration in TAA at the cellular level and functional level, 16 immune cells and 13 immune functions were incorporated to estimate the immune capacity of TAA tissues by ssGSEA. The heatmap showed the richness levels of 29 immune cells and immune functions in the TAA samples (Figure 2A). Based on the 29 immune cells and immune functions of each sample, PCA identified two distinct group-bias clusters for the TAA and control samples (Figure 2B). Compared with control group, ten immune functions (for example, inflammation-promoting, type I IFN Response, T cell costimulation, check-point, and cytolytic activity) showed significant alterations in TAA samples (Figure 2C). There were 10 types of immune cells displaying different infiltrating levels, including macrophages, neutrophils, Th1 cells, mast cells, and DCs (aDCs, pDCs) (Figure 2D). To validate the reliability of the above results, we also performed the CIBERSORT algorithm to assess the fraction of twenty-two types of immunocytes based on the characteristics of immune-related gene expression *via* bulk transcriptome profiles in TAA samples, which exhibited a significant increase in the infiltration of M2 macrophages and CD8^+^ T cells and a decreased in the infiltration of M0 macrophages and activated NK cells (Figures 2E,F). The above results demonstrate that macrophages and T cells play a central role in TAA pathogenesis, which is consistent with previous studies (15, 16). To further validate and clarify the underlying immunobiological characteristics of TAA, we examined the scRNA-seq data of TAA in the subsequent analysis.

### Single-Cell RNA Sequencing Data Preprocessing and Selection

**FIGURE 3:**
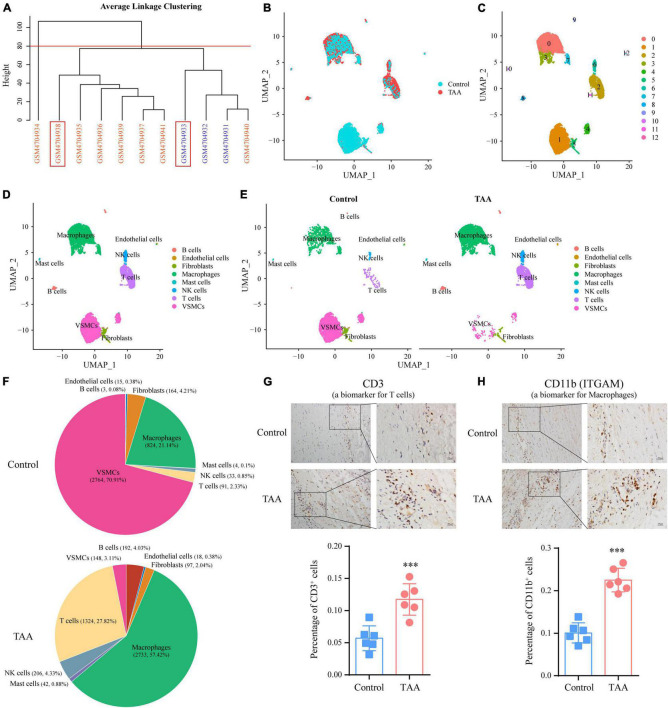
Identification of cell populations by single-cell RNA sequencing (scRNA-seq) analysis in TAA and control samples. **(A)** Sample clustering was performed based on the cellular composition of the 11 samples (8 TAA samples and three controls). Among them, GSM4704938 (TAA sample) and GSM4704933 (control sample) were chosen for further analysis. **(B)** Cell distributions in TAA and normal aortic samples are shown in a uniform manifold approximation and projection (UMAP) plot. After quality control, 4,760 and 3,898 cells from TAA and normal aortic samples, respectively, were captured for clustering analysis. **(C)** UMAP plot displaying the aggregate cells with colors denoting different clusters. **(D)** UMAP plot showing the identification of cell types for cell clusters based on cell-specific markers. **(E)** A comparison of the identified cell populations between the TAA and control groups is shown in the UMAP plot. **(F)** Pie plot exhibiting the percentages of each cell population in the control and TAA groups. **(G,H)** IHC staining showing the expression levels of CD3 (a marker of T cell; **G**) and CD11b (a marker of macrophage; **H**) in human TAA and normal aortic samples, which validated the elevation of the T cells and macrophages in TAA. ***p < 0.001; *n* = 6, Student’s *t* test.

In the present study, we reanalyzed scRNA-seq data (GSE155468) to assess the cellular composition of the thoracic aortic wall and reveal how the landscape of immune cells and their gene expression profile are altered. First, we performed individual data quality control and cell cluster identification by using the Seurat package to obtain a preliminary estimate of the cell composition of each sample (Supplementary Figures 1, 2). After characterizing each cell cluster by cell type-specific markers (Supplementary Figure 3), we examined the percentages of each cell population, which indicated significant alterations in cell composition between the two groups. However, we also noticed that the composition ratio of the same cells in different control (or TAA) samples was quite different. The heterogeneity of the different samples and the smaller sample size of the control group may limit the statistical power of this comparison. In addition, two control samples (GSM4704931 and GSM4704932) came from heart transplant recipients without aortic aneurysms, which may have exhibited molecular or cellular changes in the ascending aorta related to their cardiac disease (16). Taking the above limitations into consideration, we decided to select two representative samples to perform the analysis and display the results. Based on the clustering analysis of cellular composition from the 11 samples, we chose GSM4704938 and GSM4704933 to represent the TAA and control groups, respectively, for further analysis (Figure 3A).

### Validation of Immune Cell Infiltration in Thoracic Aortic Aneurysm by Single-Cell RNA Sequencing Data

After integrating the TAA and control data by the CAA algorithm of Seurat, unbiased cell clustering analysis was performed (Figure 3B). Similar to the separate clustering data, 13 clusters representing eight-cell lineages were identified, encompassing ECs, VSMCs, fibroblasts, macrophages, mast cells, NK cells, T cells, and B cells (Figures 3C,D). Compared with the control group, obvious changes in cell composition were observed. Such changes included a decrease in the VSMC population and expansion of immune cell populations, especially macrophages (control vs. TAA: 21.14 vs. 57.42%) and T cells (control vs. TAA: 2.33 vs. 27.82%) (Figures 3E,F), consistent with previous studies (16). In addition, we quantified the expression of CD3 (a biomarker of T cell) and ITGAM (a biomarker of macrophage) (27) to assess T cell and macrophage infiltration in TAA by IHC staining, which confirmed the results of the scRNA-seq data (Figures 3G,H). Notably, we also verified the decrease in VSMCs population and an increase in B cells by IHC staining in Supplementary Figure 4.

**FIGURE 4:**
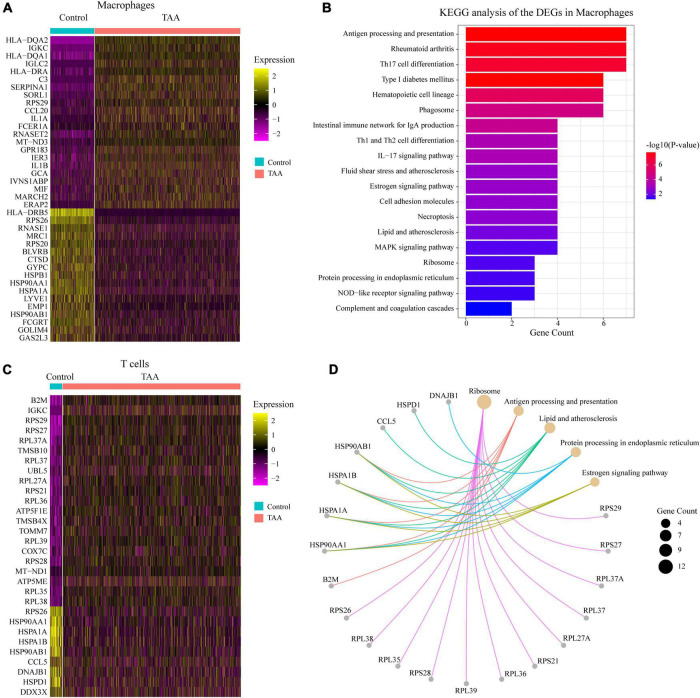
Analysis of the macrophages and T cells. **(A,C)** Heatmaps show the expression of DEGs with log_2_(FC) > 0.25 and adjusted *P*-value < 0.05, selected from macrophages **(A)** and T cells **(C)** between TAA and control samples. **(B,D)** KEGG annotations of the DEGs in macrophages **(B)** and T cells **(D)** were performed. KEGG pathways with an adjusted *P*-value < 0.05 were considered significant.

To gain more insights into the macrophages and T cells in TAA, we compared the gene expression profiles of the macrophage/T cell population between control and TAA tissues. There were 39 DEGs identified in the macrophage population with an adjusted *P*-value < 0.05 and log_2_FC > 0.25 (Figure 4A). The KEGG analysis revealed that the 39 DEGs were mainly enriched in Antigen processing and presentation, Th-cell differentiation (Th17, Th1, and Th2), Phagosome, and IL-17 signaling pathway (Figure 4B). In the T cell population, a total of 30 DEGs were screened with an adjusted *P*-value < 0.05 and log_2_FC > 0.25, which were involved in Ribosome, Antigen processing and presentation, Lipid and atherosclerosis, Protein processing in endoplasmic reticulum, Estrogen signaling pathway (Figures 4C,D). Based on the above results, we found that some pathways enriched by the DEGs of macrophages were the same as the immune-related pathways identified by the bulk transcriptome data (GSE26155). These pathways included Complement and coagulation cascades, Hematopoietic cell lineage, Rheumatoid arthritis, and Phagosome (Figures 1E, 4B), which suggests that the inflammatory responses facilitating the initiation and progression of TAA were mainly induced by macrophages. In addition, we performed pseudotime analysis for macrophages and T cells to reveal the cell transition during the biological process from normal aorta to TAA, respectively (Supplementary Figure 5). Our results showed the different subtypes (states) of macrophages and T cells between TAA and control samples. Along with trajectory progression, T cells experienced seven states from normal aorta to TAA, while macrophages experienced more states, which further validated the recruitment and differentiation of macrophages and T cells contributing to the formation of TAA. Therefore, the next questions that we should answer are: which cells recruit macrophages to the aortic wall, and how the alterations of macrophages lead to the loss of VSMCs, i.e., intercellular communications?

### Inference of Intercellular Communications in Thoracic Aortic Aneurysm

**FIGURE 5:**
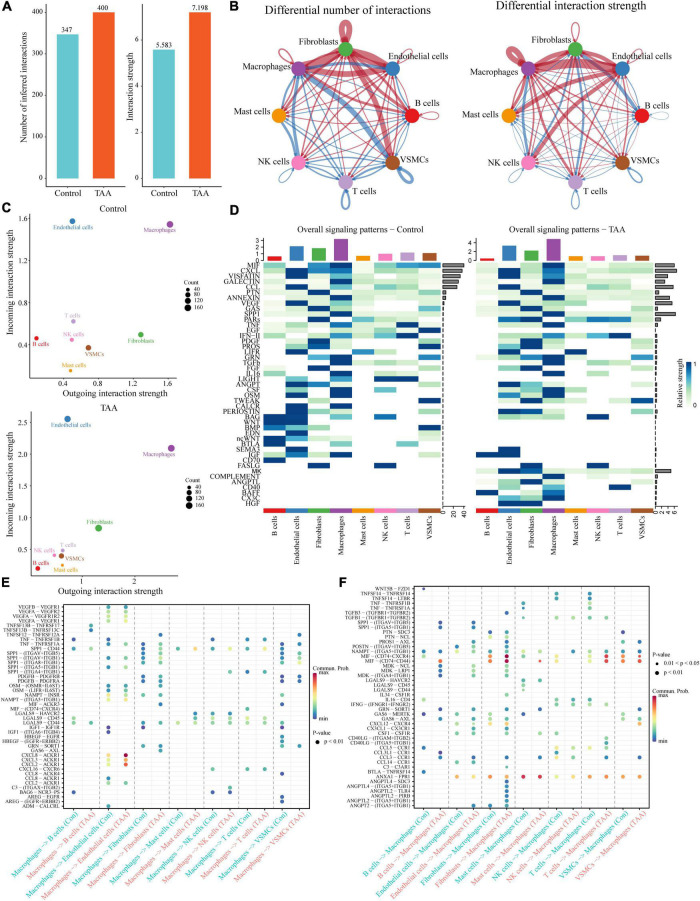
Inference of the cell-cell communications among the identified cells in TAA. **(A)** CellChat analysis indicates the increase in inferred interactions (number of interactions and interaction strength) among the cells in TAA. **(B)** Overview of the intercellular communication networks among the identified cell lineages was measured by network centrality analysis. Nodes with different colors represent different cell populations, and the red (blue) edge represents an increase (decrease) in the number of interactions or interaction strength in TAA compared with controls. **(C)** Scatter diagram showing the macrophages as the most prominent signal senders and receivers in TAA and control samples. **(D)** Heatmaps showing the relative strength of 45 signaling pathways among the eight cell populations in the overall signaling patterns. A gradual change in the color from blue to white indicates the change in the relative interaction strength from high to low. **(E,F)** The significant ligand-receptor pairs between TAA and controls are shown in dotplots, which contribute to the signaling from macrophages to other cells, including B cells, endothelial cells, fibroblasts, mast cells, NK cells, T cells, and VSMCs **(E)**, or from other cells to macrophages **(F)**. Dot color reflects communication probabilities and dot size represents computed *P*-values.

To further explore the interactions among the identified cells, the selected scRNA-seq data (GSM4704938 and GSM4704933) were used to infer intercellular communications by applying the CellChat package (29). It is well known that signaling crosstalk among different cells is essential for informing diverse cellular decisions (32). The joint analysis of CellChat provides an opportunity to discover major signal changes that might drive the pathogenesis of TAA. Compared with the controls, the inferred interactions between cells in the TAA samples increased in both the number of interactions and the strength of the interaction, especially the cell-cell communications of macrophages, fibroblasts, ECs and VSMCs (Figures 5A,B). Based on the incoming/outgoing interaction strength, the cells were jointly mapped onto a shared two-dimensional manifold, which showed that macrophages were the most prominent signal senders and receivers in TAA and controls, suggesting that macrophages play an important role in both the physiological and pathological conditions of the aorta (Figure 5C). Furthermore, CellChat detected 45 signaling pathways among the eight cell populations, including multiple inflammatory signals, such as the MIF, CXCL, GLAECTIN, CCL, ANNEXIN, TNF, IFN-II, TGFb, IL-16, LIGHT, and COMPLEMENT pathways (Figure 5D). Therefore, we focused on the intercellular communications between macrophages and other cells and the changes to the signaling pathway between TAA and control samples (Figures 5E,F).

**FIGURE 6:**
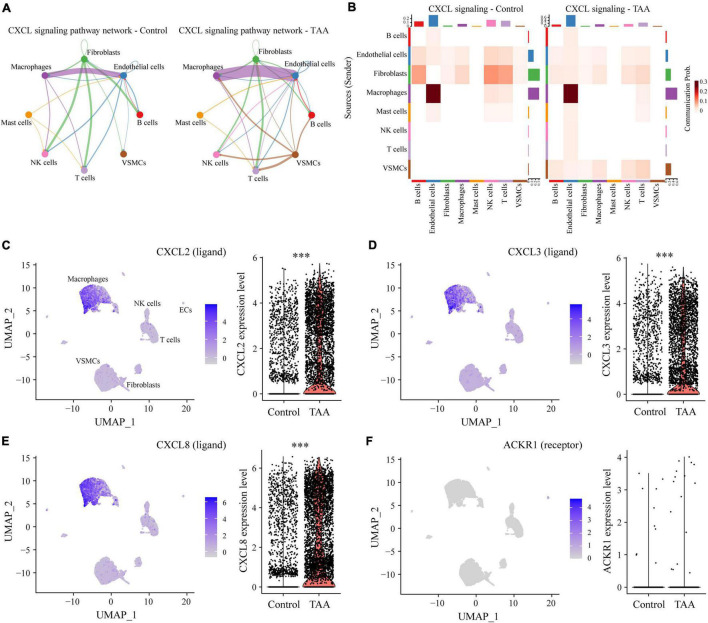
Representative signaling sent by macrophages. **(A)** Circular network plot showing the network centrality analysis of the CXCL signaling pathway. **(B)** Heatmap plot showing the communication probabilities of the CXCL signaling pathway among the cells in control and TAA samples. **(C–F)** UMAP and violin plots displaying the expression levels of CXCL2 **(C)**, CXCL3 **(D)**, CXCL8 **(E)**, and ACKR1 **(F)** in all aortic cell clusters. ***P < 0.001.

The predicted results showed that as the signaling sources, a large portion of the outgoing macrophage signaling was received by ECs, fibroblasts and VSMCs (Figure 5E). By comparing the overall communication probability from macrophages to ECs between the two groups, multiple pathways were found to be highly active in TAA. For example, network centrality analysis of the CXCL signaling pathway revealed that macrophages are the main sources of the network (Figures 6A,B). The ligands CXCL2/3/8 and their receptor ACKR1 were inferred to be the most significant signaling pathways contributing to the communication from macrophages to ECs (Figures 5E, 6). In particular, the expression levels of CXCL2, CXCL3, and CXCL8, which are mainly located in macrophages, were significantly increased in TAA (Figures 6C–E), while ACKR1 was upregulated in ECs (Figure 6F). As the signaling targets, most of the incoming macrophage signaling came from fibroblasts and ECs (Figure 5F). Specific to MIF signaling, the MIF ligand and its multisubunit receptors CD74/CD44 and CD74/CXCR4 were found to act as major signaling from fibroblasts and T cells to macrophages in TAA compared to control samples (Figure 5F and Supplementary Figure 6). The results showed that CD74, the main receptor of MIF, was significantly upregulated in TAA and almost expressed in macrophages, while the expressions of CD44 and CXCR4 were also increased in the macrophages of TAA (Supplementary Figures 6D–F). Thus, in the present study, we not only provide a comprehensive evaluation of immune cell composition in human TAA tissues but also explore the interactions between immune cells (especially macrophages) and non-immune cells. These findings improved our understanding of TAA formation and progression.

## Discussion

The progression from normal aorta to aortic dilatation to final aortic aneurysm is a multifactorial process that has only been partially revealed. The main pathological changes of TAA are medial degeneration consisting of VSMC depletion, elastic fiber fragmentation, and collagen degradation, which was originally described by Erdheim as a non-inflammatory lesion (4, 33). However, with the deepening of our understanding of TAA pathogenesis, increasing evidence shows that immune and inflammatory responses are involved in the medial degeneration that is related to dilated aortas (9, 16, 34). To explore the underlying mechanisms of immunity and inflammation in TAA, we first performed KEGG pathway analysis for the identified DEGs of the GSE26155 dataset. Unsurprisingly, multiple immune-related pathways were enriched, such as the TGF-β signaling pathway, Hematopoietic cell lineage, Leukocyte transendothelial migration, Complement and coagulation cascades, Fc gamma R-mediated phagocytosis, and Platelet activation. To further depict the immune landscape of TAA and reveal how the immunological pathways are altered, we compared the composition of immune cells between TAA and control samples by ssGSEA. The results showed that multiple types of immune cells and immune functions displayed different infiltrating levels and significant alterations in TAA. These types of altered immune cells included macrophages, T helper cells, neutrophils, DCs (aDCs and pDCs), and mast cells.

As a well-known infiltrating immunocyte, macrophages have been confirmed to be associated with the formation of TAA. Previous reports have suggested that macrophages could contribute to TAA initiation and progression by secreting MMPs and proinflammatory factors to facilitate ECM destruction and VSMC apoptosis (35). In addition, Boytard et al. (36) observed the distribution of subtypes of macrophages as the course of the abdominal aortic aneurysm (AAA) progressed. At the early stage of aortic aneurysm, M1 macrophages dominate at the site of injured aortic tissue and function in inflammatory factor expression, proteolysis, and phagocytosis. At the late stage, M2 macrophages accumulate preferentially and facilitate reparative processes, such as ECM deposition and angiogenesis (35, 36). In the present study, we found M2 macrophages rather than M1 macrophages increased significantly in TAA (Figure 2F), which maybe because the samples in GSE26155 were largely the elderly degenerative TAAs within the advanced stage. Notably, whether macrophage subsets have similar evolution in TAA compared to AAA requires further experimental verification in the future.

Recently, an intriguing type of VSMC with a macrophage-like phenotype was identified as macrophage-like VSMCs, which acquired characteristics reminiscent of immune cells but continued to express markers of the original VSMCs (37). However, it’s more appropriate to identify them as macrophages rather than VSMCs. The macrophage-like VSMCs suppress the expression of classic VSMC markers (such as SM22a, ACTA2, and MYH11), and turn on the expression of multiple macrophage markers, including CD68, CD11b, and LGALS3 (37–39). It’s reported that macrophage-like VSMCs were involved in chronic inflammatory processes by producing different cytokines (such as IL1b, IL8, IL6, and CCL2) and various adhesion molecules (37). In turn, these cytokines further recruited immune cells and promoted various biological processes. Chen et al. (38) concentrated on the VSMC reprogramming in aortic aneurysm and identified a modulated VSMC with a macrophage-like phenotype by using cell fate tracing with the CreER*^T2^*-loxP system and scRNA-seq analysis. Furthermore, they found that the key driver of this phenotype switch appeared to be a large increase in Klf4 expression and VSMC-specific Klf4 knockout largely prevented aneurysm development in this model. In addition, an increasing body of VSMC lineage tracing studies have revealed VSMCs undergoing remarkable changes in phenotype during development of atherosclerosis (39–42). It’s demonstrated that VSMCs in atherosclerosis expressed increased Klf4 and the transition to macrophage-like state was Klf4-dependent by utilizing VSMC-specific lineage tracing mice ± simultaneous VSMC-specific conditional knockout of Klf4 (41). However, there is no study using cell lineage tracking to investigate the process of phenotypic switching or cellular dedifferentiation in TAA. Whether macrophage-like VSMCs also exist in TAA, and which regulator is responsible for the transition, and what roles the special VSMCs play in initiation and progression of TAA all need further research to elaborate.

In addition to macrophages, T lymphocytes are frequently observed in TAA samples by IHC staining and flow cytometry (9). A number of previous studies reported that T cells are significantly increased in samples from patients with TAA compared with control aortas (9, 34), which is consistent with our results. To assess the infiltration of T cells in aortic walls, Wang et al. (43) detected CD3-positive cells in TAA samples and found accumulating CD3-positive cells predominantly in the adventitia and media of TAA, which implies that T cells might migrate from the adventitia into the media of the aortas. We demonstrated that the aortic walls of TAA patients display significantly more T cells than those in normal aortas. However, it should be noted that different T cell subsets have complex effects during the inflammatory response and might even contradict each other (44). For example, Th1 and Th2 (subsets of naïve CD4-positive T cells) are characterized by the production of IFN-γ and IL-4, respectively (45). Several studies reported that Th1 cells, as the predominant type of CD4^+^ T cells in TAA, positively correlated with aortic expansion in aneurysm patients (46–48). Upregulation of IFN-γ produced by Th1 cells significantly correlated with both the outward vascular remodeling and intimal expansion of TAA (46). After examining the immunocytes of TAA tissues, Tanimura et al. reported that stimulation of the Th1/IFN-γ system could lead to aortic aneurysm formation by inhibiting Treg cell proliferation (48). In contrast, other groups have found that CD4^+^ T cells in aortic aneurysms are predominantly IL-4-producing Th2 cells. Ntika et al. (49) reported that no change was observed in the Th1 cytokines in the TAA group; however, cytokines associated with Th2 cells, such as IL-4, IL-5, and IL-10, were significantly altered in TAA patients compared to the control individuals.

Currently, in addition to some algorithms for assessing the infiltration of immune cells in tissues based on bulk transcriptome profiles, scRNA-seq provides an opportunity for us to reveal complex immune cell populations (50, 51). To further validate the above-described findings based on the ssGSEA algorithm, we not only analyzed scRNA-seq data but also detected the infiltration of macrophages and T cells in human TAA samples by IHC staining. These results also revealed the expansion of macrophages and T cells. As the most suitable technology for revealing the cellular composition of tissues and gene expression profiles at the single-cell level, various studies have used the scRNA-seq technique to delineate the cellular heterogeneity of cardiovascular diseases. Zhao et al. (52) and Yang et al. (53) performed scRNA-seq on mouse AAA models to reveal the cellular heterogeneity of AAA. After integrative analysis of scRNA-seq data from ascending aortic aneurysm tissue in Marfan syndrome mice and humans, Pedroza et al. characterized the disease-specific signature of modulated VSMCs, a distinct cluster of cell populations, which might be driven by TGF-β signaling and Klf4 overexpression (54). However, at present, there is just one study that uses scRNA-seq analysis to uncover the cellular and molecular landscape of human TAA tissues without heritable diseases (16). In general, they identified 10 major cell types in human ascending aortic tissue, most of which were the same as our results. Compared with the control tissues, TAA tissues had fewer non-immune cells (such as VSMCs) and more immune cells, especially T lymphocytes. Their differential gene expression data suggested the presence of extensive mitochondrial dysfunction in TAA tissues.

Notably, scRNA-seq data inherently contain transcriptomic profile information that could be used to infer intercellular communications. The CellChat package was developed to predict and visualize cell-cell communications from scRNA-seq data based on the known structural composition of ligand-receptor interactions, as well as stimulatory and inhibitory membrane-bound coreceptors (29). By applying CellChat, macrophages were determined to be the most prominent signal senders and receivers in the TAA and control samples. These findings suggest that macrophages play critical roles in both the physiological and pathological conditions of the aorta, which was consistent with the current state of knowledge (35, 55). Whether in normal aorta or TAA tissues, the cells that interact most frequently are macrophages, ECs, fibroblasts, and VSMCs, which implies that cross-talk between these cells is essential for maintaining physiological homeostasis in the aorta, and dysfunction of the intercellular interactions can lead to the aortic disease. Unsurprisingly, the interaction between macrophages and ECs (or fibroblasts) is significantly enhanced, while the interaction with VSMCs is reduced in TAA. Notably, the transwell migration assays of VSMCs and macrophages showed that rs12455792 variant of *SMAD4* gene in VSMCs significantly increased macrophages recruitment *via* activated TGF-β signaling pathway (56). In our study, we observed the activation of TGF-β signal from NK cells and T cells to macrophages (Figure 5F). Saito et al. (57) demonstrated that the endothelium played important roles in triggering macrophage infiltration and inflammation in the aorta, resulting in the vascular remodeling and aneurysm formation through intracellular NF-kB signal interaction. In addition, monocyte recruitment to the aorta requires the upregulation of chemoattractant or activating cytokines and adhesion molecules, such as Cyr61, ICAM-1/2, VCAM-1, and MCP-1, that are produced and secreted by ECs, fibroblasts, and other cell types (58). Zhou et al. (59) found that myelin debris significantly increased endothelial secretion of MCP-1 and other proinflammatory mediators (for example IL-4 and IL-6), which may contribute to macrophage infiltration in microvessels. In contrast, Gitzin et al. (60) demonstrated that following carotid injury, patrolling monocytes were recruited to the endothelium at wound sites to promote EC proliferation and tissue repair. Combining the above results and the anatomy of the aorta, we hypothesize that ECs and fibroblasts recruit macrophages by enhanced intercellular communications *via* CXCL and MIF signals. The recruitment of macrophages to the aortic wall is the primary culprit of VSMC loss, which ultimately results in aortic dilatation and rupture.

There is a limitation in our study. Based on the detailed information of TAA samples, we noticed that the mean age of the TAA patients in the GSE26155 dataset was 61.5 years, and the mean age of the TAA patients in the GSE155468 dataset was approximately 70 years. In addition, these TAA samples were homogenous, and none had hereditary diseases, such as Marfan syndrome, Loeys–Dietz syndrome or bicuspid aortic valve malformation. Taking these factors into consideration, we assumed that the predominant form of TAA in this cohort was the degenerative form of non-syndromic TAA. Therefore, the conclusions of our study might be limited to degenerative non-syndromic TAA. A further study containing different TAA forms is needed to reveal the representative landscape of the immune microenvironment in the future.

## Conclusion

In conclusion, after characterizing the landscape of the immune microenvironment in TAA by various immune-related algorithms, we found that the infiltration of macrophages and T cells was mainly responsible for TAA formation among the immune cells. In particular, as macrophages are the most prominent signal senders and receivers of the aortic tissue, they play an important role in both the physiological and pathological conditions of the aorta. These immune cells might help to further provide novel insights into candidate targets of prevention and immunotherapy for TAA patients.

## Data Availability Statement

The datasets presented in this study can be found in online repositories. The names of the repository/repositories and accession number(s) can be found in the article/Supplementary Material.

## Ethics Statement

The studies involving human participants were reviewed and approved by the Ethics Committee of Tongji Hospital, Tongji Medical College, Huazhong University of Science and Technology. The patients/participants provided their written informed consent to participate in this study.

## Author Contributions

XW and D-SJ designed and directed the entire study and critically reviewed the manuscript. QW and XG performed analyses of sequencing data. BH and XF performed IHC staining experiments and statistical analyses. XF and Z-MF provided clinical samples and data. QW wrote the manuscript. All authors contributed to the article and approved the submitted version.

## Conflict of Interest

The authors declare that the research was conducted in the absence of any commercial or financial relationships that could be construed as a potential conflict of interest.

## Publisher’s Note

All claims expressed in this article are solely those of the authors and do not necessarily represent those of their affiliated organizations, or those of the publisher, the editors and the reviewers. Any product that may be evaluated in this article, or claim that may be made by its manufacturer, is not guaranteed or endorsed by the publisher.

## Footnotes

1
http://www.ncbi.nlm.nih.gov/geo/

2
https://cibersort.stanford.edu

3
http://www.cellchat.org/
