# Supplementary material for: Integrating Bulk Transcriptome and Single-Cell RNA Sequencing Data Reveals the Landscape of the Immune Microenvironment in Thoracic Aortic Aneurysms
Source: Front Cardiovasc Med. 2022 Apr 7;9:846421. doi: 10.3389/fcvm.2022.846421 (PMC9021420; doi:10.3389/fcvm.2022.846421)
Supplement: Supplementary file 1 [file Data_Sheet_1.pdf]

Integrating Bulk Transcriptome and Single-cell RNA Sequencing Data Reveals the Landscape of the Immune Microenvironment in Thoracic Aortic Aneurysms

Supplementary Figures

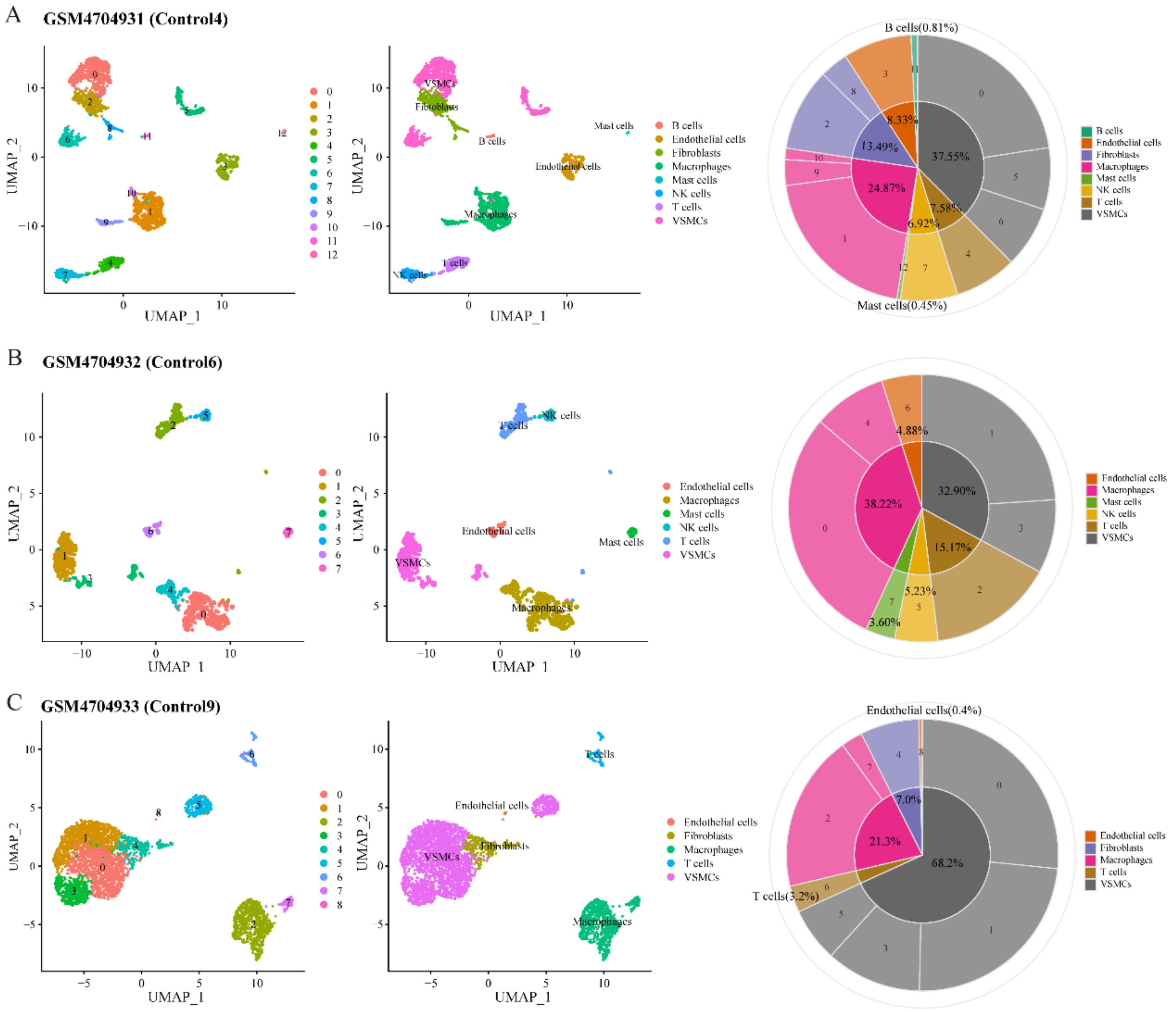

Figure S1. Separate analysis of each control scRNA-seq sample. (**A**) GSM4704931; (**B**) GSM4704932; (**C**) GSM4704933. For each panel: a UMAP plot displaying the aggregate cells with colors denoting different clusters on the left; a UMAP plot showing the identification of cell types for cell clusters based on cell-specific markers in the middle; and a Pie plot exhibiting the percentages of each cell population in the control group.

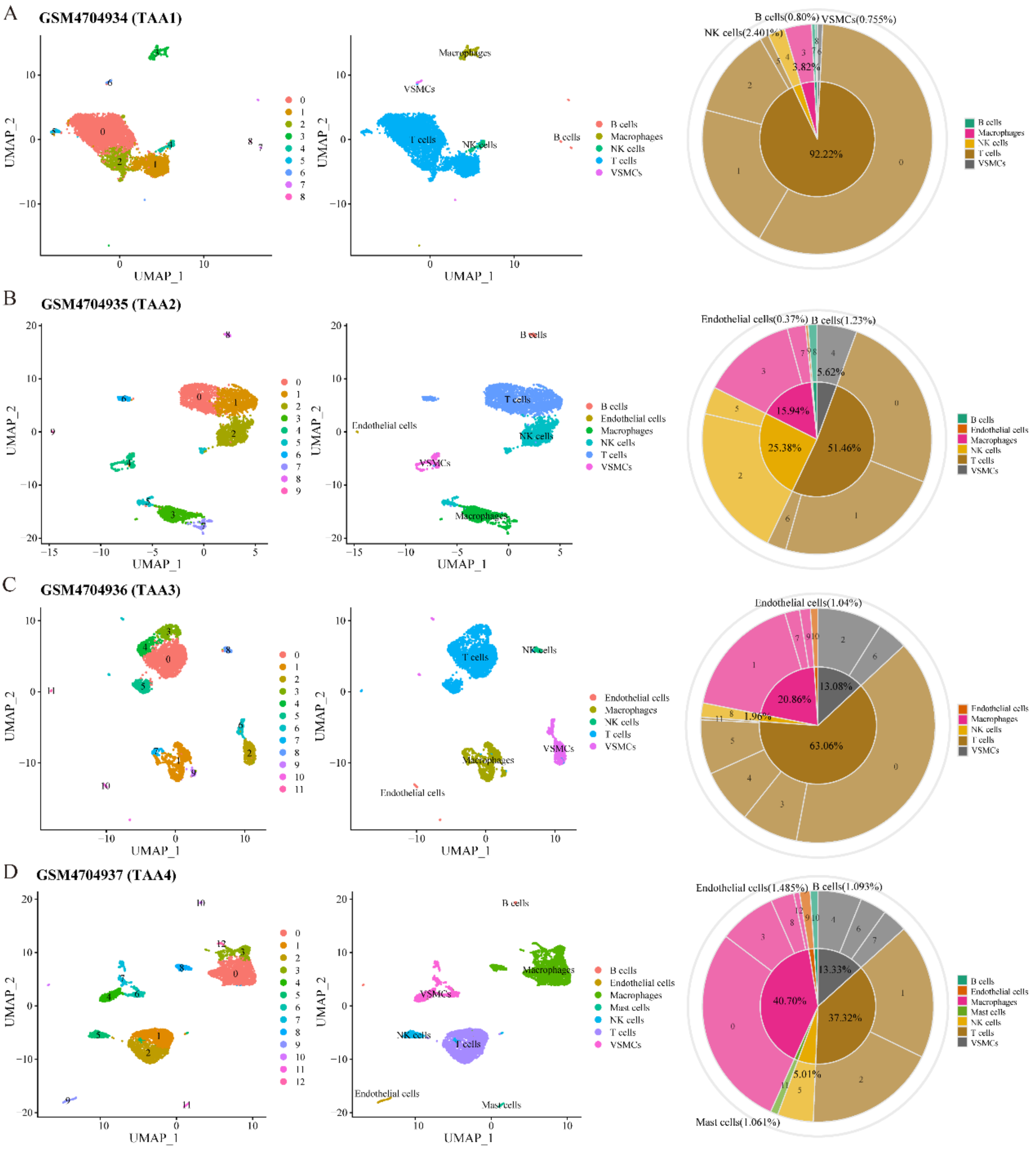
A
GSM4704934 (TAA1)
UMAP_1
UMAP_2
Macrophages
VSMCs
T cells
NK cells
B cells
B cells
Macrophages
NK cells
T cells
VSMCs
B cells(0.80%)
VSMCs(0.755%)
NK cells(2.401%)
3.82%
92.22%
B
GSM4704935 (TAA2)
UMAP_1
UMAP_2
B cells
T cells
Endothelial cells
NK cells
VSMCs
Macrophages
B cells
Endothelial cells
Macrophages
NK cells
T cells
VSMCs
Endothelial cells(0.37%)
B cells(1.23%)
5.62%
15.94%
25.38%
51.46%
C
GSM4704936 (TAA3)
UMAP_1
UMAP_2
T cells
NK cells
VSMCs
Macrophages
Endothelial cells
Endothelial cells
Macrophages
NK cells
T cells
VSMCs
Endothelial cells(1.04%)
13.08%
20.86%
1.96%
63.06%
D
GSM4704937 (TAA4)
UMAP_1
UMAP_2
B cells
Macrophages
VSMCs
NK cells
T cells
Endothelial cells
Mast cells
B cells
Endothelial cells
Macrophages
Mast cells
NK cells
T cells
VSMCs
Endothelial cells(1.485%)
B cells(1.093%)
13.33%
40.70%
37.32%
5.01%
Mast cells(1.061%)

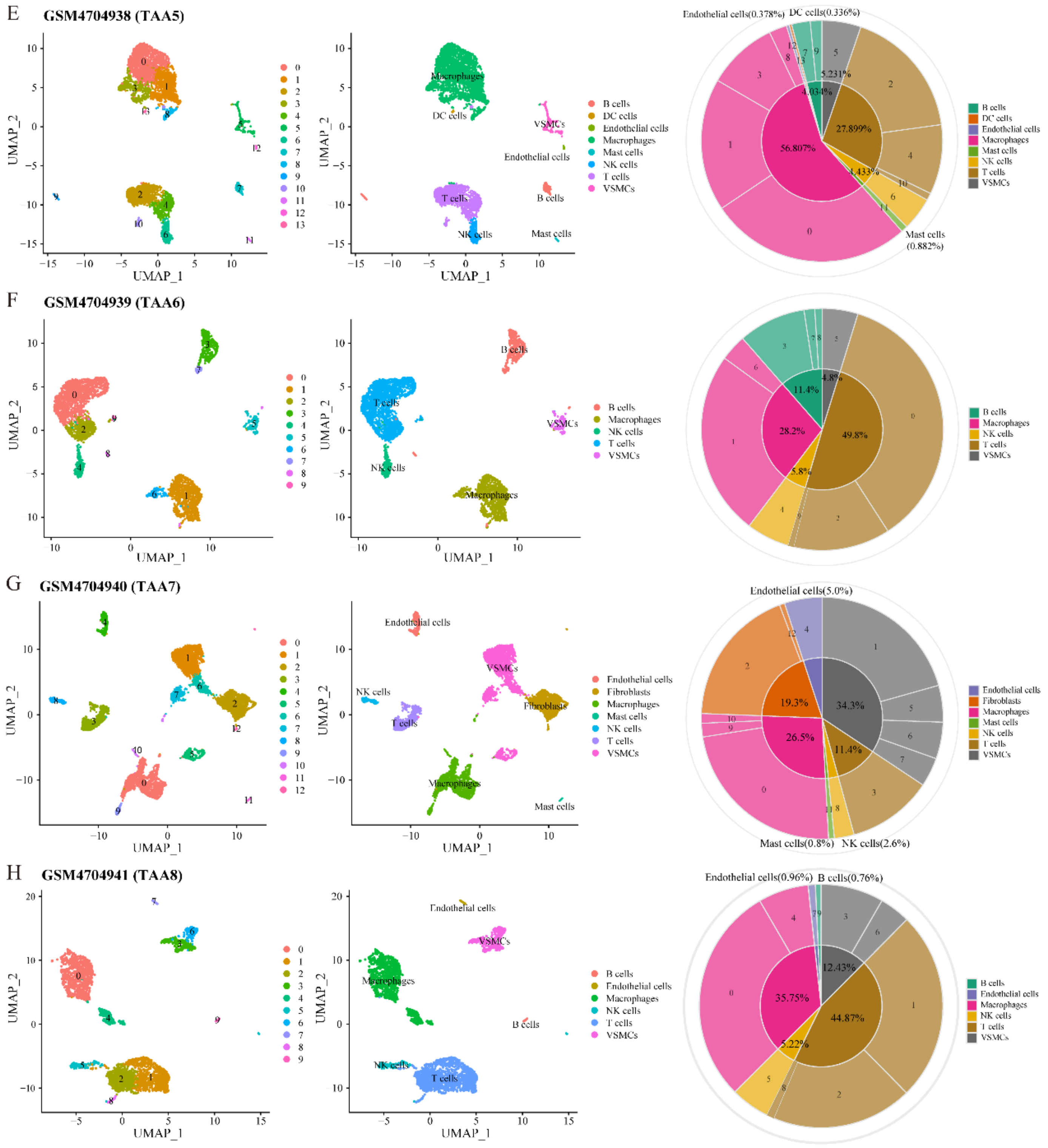

Figure S2. Separate analysis of each TAA scRNA-seq sample. (**A**) GSM4704934; (**B**) GSM4704935; (**C**) GSM4704936; (**D**) GSM4704937; (**E**) GSM4704938; (**F**) GSM4704939; (**G**) GSM4704940; (**H**) GSM4704941. For each panel: a UMAP plot displaying the aggregate cells with colors denoting different clusters on the left; a UMAP plot showing the identification of cell types for cell clusters based on cell-specific markers in the middle; and a Pie plot exhibiting the percentages of each cell population in the TAA group.

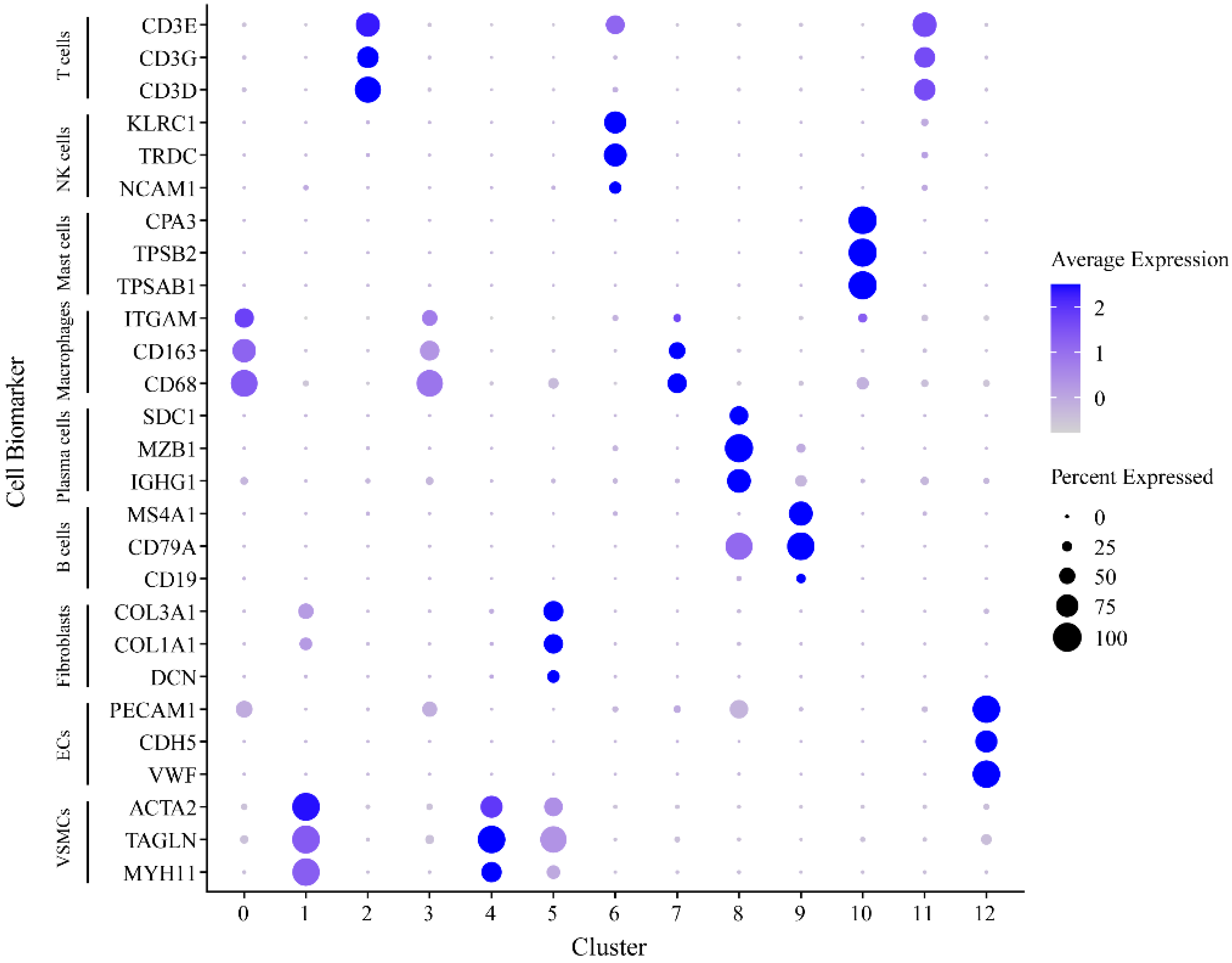

Figure S3. Dotplot showing the cell-specific markers for each cluster and lineage in all aortic cell clusters. The size of the dot represents the percentage of cells expressing each gene, while the dot color indicates the expression level.

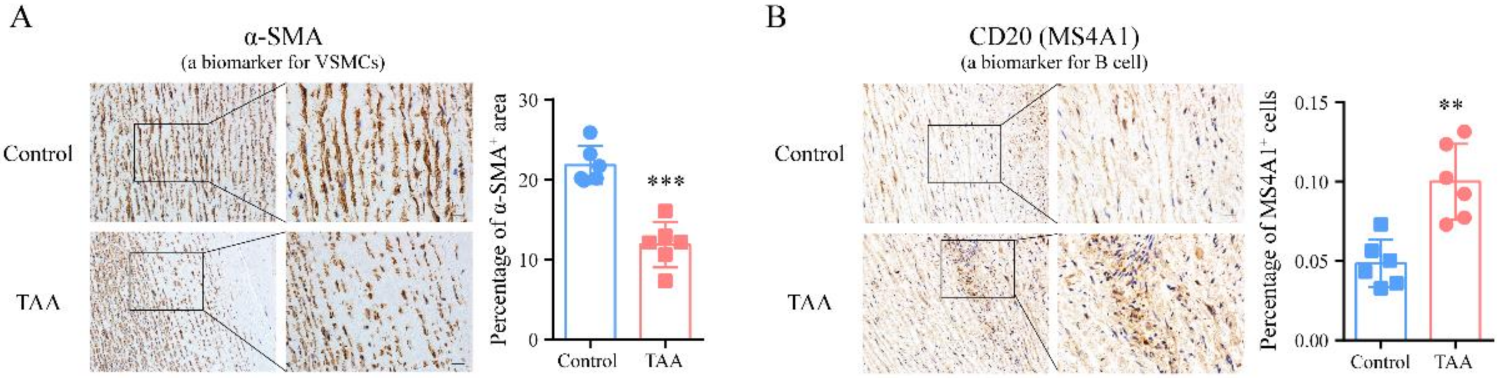

Figure S4. IHC staining showing the expression levels of α-SMA (a marker of VSMCs; A) and CD20 (a marker of B cells; B) in human TAA and normal aortic samples, which validated the decrease of VSMCs and elevation of the B cells in TAA. $^{**}p < 0.01$; $^{***}p < 0.001$, $n = 6$, Student's t test.

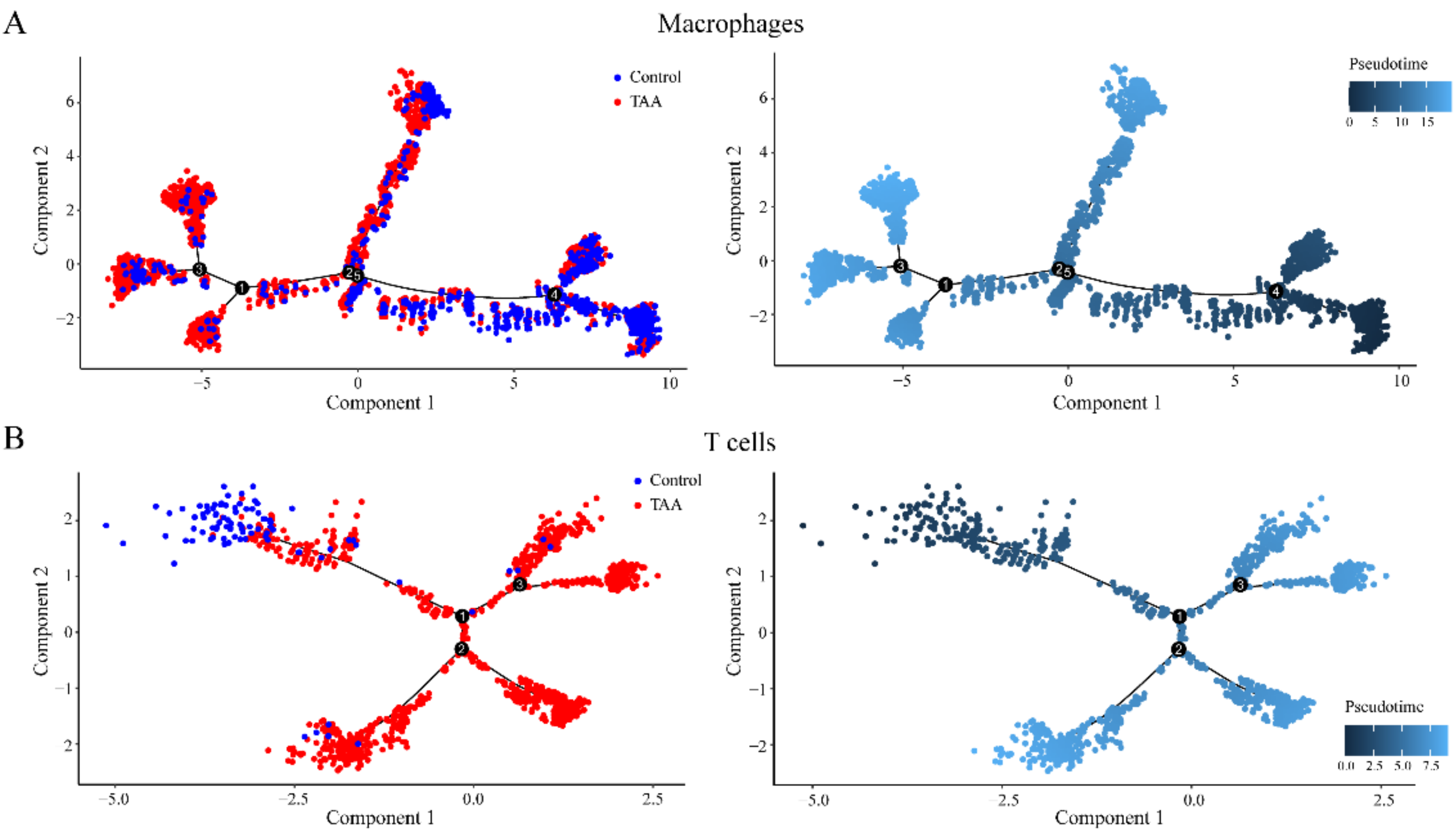

Figure S5. **Pseudotime trajectory analysis of macrophages and T cells**. (**A**) Simulation of the differentiation trajectory of macrophages from control to TAA (left panel) and pseudotime trajectory transition (right panel). (**B**) Simulation of the differentiation trajectory of T cells from control to TAA (left panel) and pseudotime trajectory transition (right panel). Pseudotime trajectory analysis reveals the transition states of macrophages (T cells) undergoing during TAA formation from normal aorta. The numbers in the black circles represent nodes in the trajectory analysis that determine the different cell states. The different colors on the left panel represent control and TAA states, and the colors on the right panel represent the order of time from dark to light.

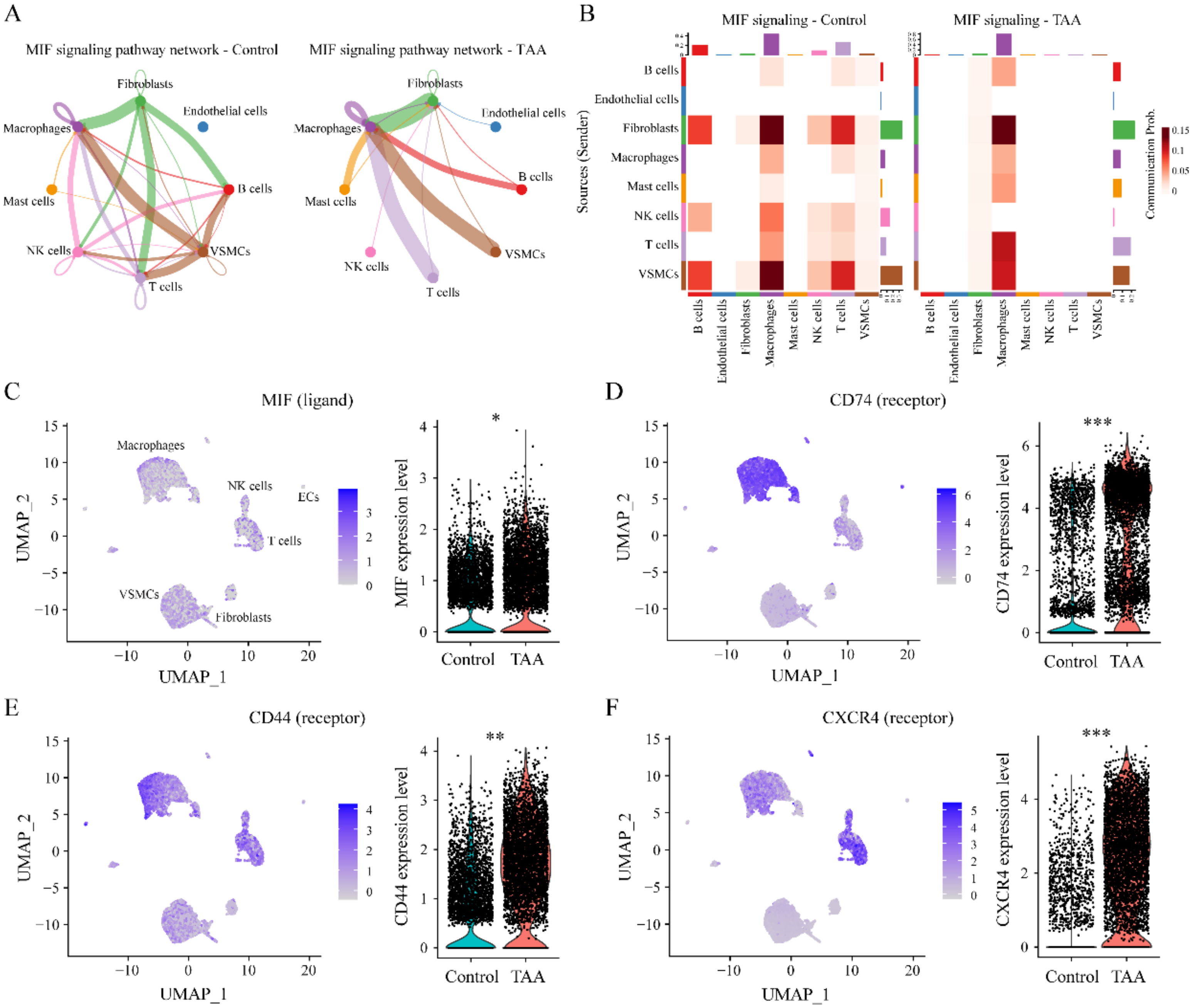

Figure S6. Representative signaling received by macrophages. (**A**) Circular network plot showing the network centrality analysis of the MIF signaling pathway. (**B**) Heatmap plot showing the communication probabilities of the MIF signaling pathway among the cells in control and TAA samples. (**C-F**) UMAP and violin plots displaying the expression levels of MIF (C), CD74 (D), CD44 (E), and CXCR4 (F) in all aortic cell clusters. *P < 0.05; **P < 0.01; ***P < 0.001.

Supplementary Table

Supplementary Table 1. Detailed information of aortic samples used in the present study

Sample	Gender	Age	Location	Diameter(mm)	Hereditary diseases	Dissection or Rupture	Reference
Control 1-6			Normal aorta obtained from heart transplant donor				—
TAA-1	male	59	Ascending aorta	53.41	No	No	—
TAA-2	mae	66	Ascending aorta	51.87	No	No	—
TAA-3	male	48	Ascending aorta	47.08	No	No	—
TAA-4	male	66	Ascending aorta	141.65	No	Yes	—
TAA-5	male	63	Arch of the aorta	57.63	No	No	—
TAA-6	female	74	Ascending aorta	71	No	No	—
GSM4704938	female	75	Root of aorta	58	No	—	16
GSM4704933	female	62	Normal aorta	22	—	—	16
GSE26155(n=13)			Normal aorta obtained from heart/lung transplant donor				15
GSE26155(n=22)	9 female/13 male	61.5 ± 15	TAA	53.6 ± 7.5	No	—	15

Reference

15. Folkersen L, Wågsäter D, Paloschi V, Jackson V, Petrini J, Kurtovic S, et al. Unraveling divergent gene expression profiles in bicuspid and tricuspid aortic valve patients with thoracic aortic dilatation: the ASAP study. *Molecular medicine (Cambridge, Mass)* (2011) 17(11-12):1365-73.

16. Li Y, Ren P, Dawson A, Vasquez HG, Ageedi W, Zhang C, et al. Single-Cell Transcriptome Analysis Reveals Dynamic Cell Populations and Differential Gene Expression Patterns in Control and Aneurysmal Human Aortic Tissue. *Circulation* (2020) 142(14):1374-88.
